# Lobelia Lakes’ Vegetation and Its Photosynthesis Pathways Concerning Water Parameters and the Stable Carbon Isotopic Composition of Plants’ Organic Matter

**DOI:** 10.3390/plants13172529

**Published:** 2024-09-09

**Authors:** Eugeniusz Pronin, Krzysztof Banaś, Rafał Chmara, Rafał Ronowski, Marek Merdalski, Anne-Lise Santoni, Olivier Mathieu

**Affiliations:** 1 Department of Plant Ecology, Faculty of Biology, University of Gdansk, 80-309 Gdansk, Poland; krzysztof.banas@ug.edu.pl (K.B.); rafal.chmara@ug.edu.pl (R.C.); rafal.ronowski@ug.edu.pl (R.R.); marek.merdalski@ug.edu.pl (M.M.); 2 Biogéosciences, UMR 6282 CNRS, Université Bourgogne Franche-Comté, F-21000 Dijon, France; anne-lise.santoni@u-bourgogne.fr (A.-L.S.); olivier.mathieu@u-bourgogne.fr (O.M.)

**Keywords:** *δ*^13^C, macrophytes, organic matter, water conditions, softwater, photosynthesis, CCM, CAM, C3

## Abstract

Most of the aquatic vegetation produces organic substances via the C3 photosynthetic pathway (mosses, isoetids—*Lobelia dortmanna* L., *Luronium natans* (L.) Raf., and vascular plants) or Crassulacean acid metabolism (CAM, e.g., *Littorella uniflora* (L.) Asch. and *Isoëtes lacustris* L.) or by their ability to use HCO_3_^−^ via carbon concentration mechanisms (CCMs—some elodeids and charophytes). Differentiating these predominant photosynthetic pathways in aquatic vegetation based on their organic matter (OM) carbon stable isotopes (*δ*^13^C_ORG_) is a complex task, in contrast to terrestrial plants. This study investigates the OM deposition, characterized by *δ*^13^C_ORG_ values in 10 macrophyte species with different photosynthetic pathways (C3, CAM, and CCM) collected from 14 softwater Lobelia lakes in northern Poland. The higher *δ*^13^C_ORG_ values distinguish the CCM group, indicating their use of ^13^C-enriched HCO_3_¯ in photosynthesis. CAM species show slightly higher *δ*^13^C_ORG_ values than C3, particularly in lower pH lakes. Principal component analysis of isotopic and environmental data did not yield clear distinctions by the groups, but still, they significantly differ in light of analyzed parameters and isotopic signals (PRMANOVA = 5.08, *p* < 0.01; K-W H = 27.01, *p* < 0.001). The first two PCA dimensions showed that the water pH and Ca^2+^ concentration positively influenced *δ*^13^C values. The influence of light conditions on *δ*^13^C_ORG_ values revealed by third PCA components seems to also be important. In summary, northern Polish Lobelia lakes serve as a key differentiation point between vegetation employing CCMs and those relying on C3/CAM photosynthesis without HCO_3_^−^ utilization, providing insights into transitions in plant communities within these ecosystems.

## 1. Introduction

The stable isotopic values of carbon (*δ*^13^C) in aquatic plants exhibit a wide range across different aquatic ecosystems (−50‰ to 0.4‰) [1,2]. Numerous studies suggest that these isotopic differences might be related to multiple variables of water conditions, such as pH and nutrient concentration [3,4,5,6,7,8,9,10,11,12]. Moreover, terrestrial and aquatic plants usually differ in their utilization of the photosynthetic pathway, which also plays a crucial role in carbon fractionation. Typically, terrestrial C3 plants have ^12^C-enriched *δ*^13^C values compared with C4 Crassulacean acid metabolism (CAM) photosynthetic plants [13]. However, limited attention has been given to these differences in aquatic plants, especially submerged aquatic species, due to the scarcity of the representation of C4 and CAM photosynthesis pathways and the relatively lower importance of these types of plants (C4 weeds as sorghum, proso millet, and corn are food sources; thus, the focus on the study related to them is higher than submerged plants) [14]. Only limited research focused on CAM and C3 aquatic plant’s isotopic data comparison [15,16,17] and C3 plants with and without carbon compensation mechanisms (CCM, [18]). This limited number of studies focusing on comparisons of different aquatic plants’ photosynthesis pathways is probably related to the limited occurrence of representative species, e.g., CAM *Isoëtes* and *Littorella* species, which could be found only in the specific environmental conditions, especially of water and sediment [19]. One among the limited aquatic CAM plants is *Littorella uniflora* (L.) Asch., which stands out as an amphibious species with high ecological plasticity and is used in numerous research experiments [20,21,22,23].

Physical and chemical conditions significantly impact the values of *δ*^13^C in water. The source of carbon used in photosynthesis plays a crucial role in determining the stable isotope composition of aquatic plant’s organic matter (OM). The pH of the water, however, is a key factor that influences the availability of different forms of carbon. In water, with a pH range of around 4.5–6.0, the dominant form of carbon is carbon dioxide (CO_2_), which is enriched in the ^12^C isotope. At pH values from 6.0 to 9.0, the dominant form is bicarbonate (HCO_3_¯), which is enriched in the ^13^C isotope by about 8–12‰ relative to CO_2_. The third form of inorganic carbon in water is carbonate ion (CO_3_^2−^), which dominates at a pH above 9. Plants with C3 and CAM photosynthetic pathways mainly use CO_2_ as a source for their photosynthesis. However, only some aquatic groups of plants have developed mechanisms for using HCO_3_^−^ for photosynthesis [24,25]. This primarily occurs under the conditions of CO_2_ deficiency, which is the preferred carbon source for all plant groups. This mechanism, named CO_2_ compensation mechanisms (CCM), occurs in many submerged aquatic plants, especially flowering plants from the genus of *Elodea*, *Myriophyllum*, and *Potamogeton* [26]. The efficiency of the CCM process varies between species. For instance, in the case of *Elodea canadensis* Michx, it is more effective than *Myriophyllum alterniflorum* DC [27,28].

The *δ*^13^C values can be significantly influenced by the structure of vegetation, thereby indirectly impacting the differentiation of inorganic carbon forms within water. This dependency occurs through increased pH caused by intense photosynthesis and substantial depletion of ^12^C in the water [11]. Such alterations in the pH, particularly notable in specific softwater lakes (with low Ca^2+^ ions concentration), are characterized by unique vegetation from the isoetid group of plants (i.e., *Lobelia dortmanna* L., *Luronium natans* (L.) Raf. *L. uniflora* and *Isoëtes lacustris* L.), referred to as Lobelia lakes or softwater lakes with isoetids [11,19]. These lakes have a low buffering capacity with low calcium and other ion concentrations, resulting in low electrical conductivity. Thus, due to intensive photosynthetic activities, especially within these lakes, plants are prompted to incorporate more ^13^C, which potentially reflects in the isotopic composition of the plant’s OM.

Moreover, specific vegetation in the Lobelia lakes is characterized by its sensitivity to environmental changes, particularly alkalization, acidification with the humification processes, and eutrophication [29,30,31]. Isoetids are perennial plants that outcompete phytoplankton in lakes or littoral areas, forming dense communities that extensively cover the lake bed [19]. In contrast, charophytes and elodeids do not develop expansive monospecific communities in Lobelia lakes [29].

Hence, this research aims to investigate the applicability of the stable carbon isotopic values of plants’ organic matter (*δ*^13^C_ORG_) values in distinguishing the photosynthetic pathways and mechanisms adopted by prevalent aquatic vegetation in northern Polish Lobelia lakes. In addition, we aimed to recognize the connection between the *δ*^13^C_ORG_ values of three divided groups, i.e., C3, CAM, and CCM, while considering the carbon availability affected by pH fluctuation and other water and sediment variables. 

This research has significant potential applications and implications. Investigating the *δ*^13^C_ORG_ values in northern Polish Lobelia lakes could offer insights into the photosynthetic pathways and mechanisms of aquatic vegetation, which are vital for understanding carbon cycling in these ecosystems. Additionally, distinguishing between C3, CAM, and CCM photosynthetic types based on *δ*^13^C_ORG_ values can help assess the impact of environmental changes, such as pH fluctuations and other water and sediment variables, on aquatic plant metabolism. This could ultimately aid in managing and conserving these unique ecosystems by providing a clearer picture of how aquatic plants adapt to environmental stressors.

## 2. Results

### 2.1. δ^13^C_ORG_ Values of Plants in the Light of the Photosynthetic Group and Mechanisms

Our results showed differences between the three investigated groups for *δ*^13^C_ORG_ (K-W H = 27.01, *p* < 0.001). The highest values *δ*^13^C_ORG_ were exhibited by the CCM group, which was as expected due to their ability to use ^13^C-enriched HCO_3_^−^. However, within this group, the highest variability of the obtained results was related to the different ecological spectra of plants included in this group (i.e., charophytes and flowering vascular plants from the elodeids group). 

**Figure 1:**
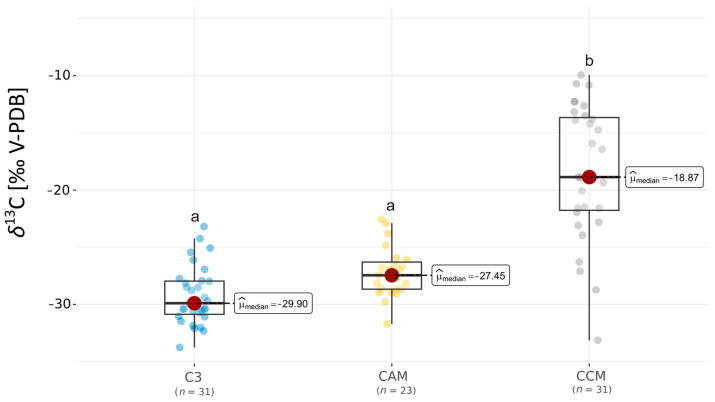
Comparison of δ^13^C values of plants’ OM investigated from a species group concerning their photosynthesis pathways and carbon acquisition mechanism (CCM). The lowercase letters above boxplots, if they differ, indicate the statistical significance of the Dunn post hoc (*p* < 0.05) after the Kruskal–Wallis test.

Conversely, the C3 group exhibited the lowest values based on the median results of *δ*^13^C_ORG_ (Figure 1). However, in the CAM group, we observed relatively low values of *δ*^13^C_ORG,_ with a median close to this reported for C3 plants (Figure 1). In general, the variability of obtained *δ*^13^C_ORG_ in the C3 and CAM groups was lower compared with the CCM group (Figure 1). Additionally, the differences between the C3 and CAM groups with the CCM group were statistically significant (Dunn posthoc test *p* < 0.05). 

### 2.2. Relationships between Water Physicochemical Variables and the δ^13^C_ORG_ of Plant and Sediments OM

**Figure 2:**
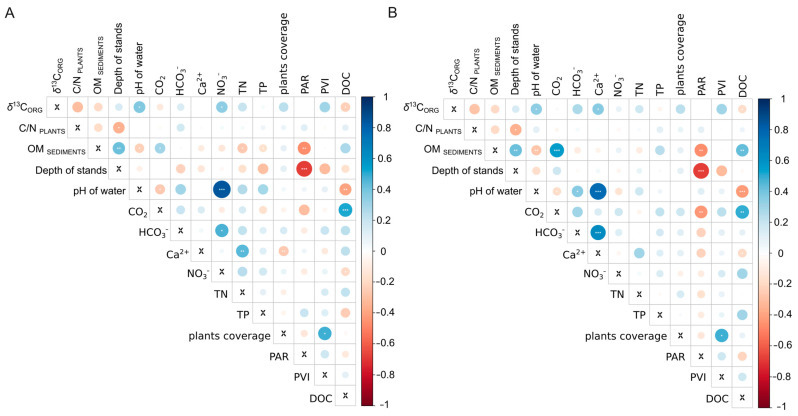
Spearman rank correlations heat map of (**A**) ambient waters variables and (**B**) sediment water variables. OM _SEDIMENTS_—% of the OM in the sediments, TN—total nitrogen and TP—total phosphorus, DOC—dissolved organic carbon, PAR—photosynthetic active radiation, and PVI—percentage volume infested by plants. * *p* < 0.05, ** *p* < 0.01, *** *p* < 0.001.

During our comparisons of δ^13^C_ORG_ of plants with other investigated parameters, we identified several significant relationships both for parameters measured in ambient and above sediment water (Figure 2A,B). Among them, the most important relationships were found between δ^13^C_ORG_ and the pH, Ca^2+^ (Figure 2A,B), and only for ambient water with NO_3_^−^. We also noted a moderate negative relationship between δ^13^C_ORG_ and DOC, namely the dissolved organic carbon concentration (Figure 2A,B). 

**Figure 3:**
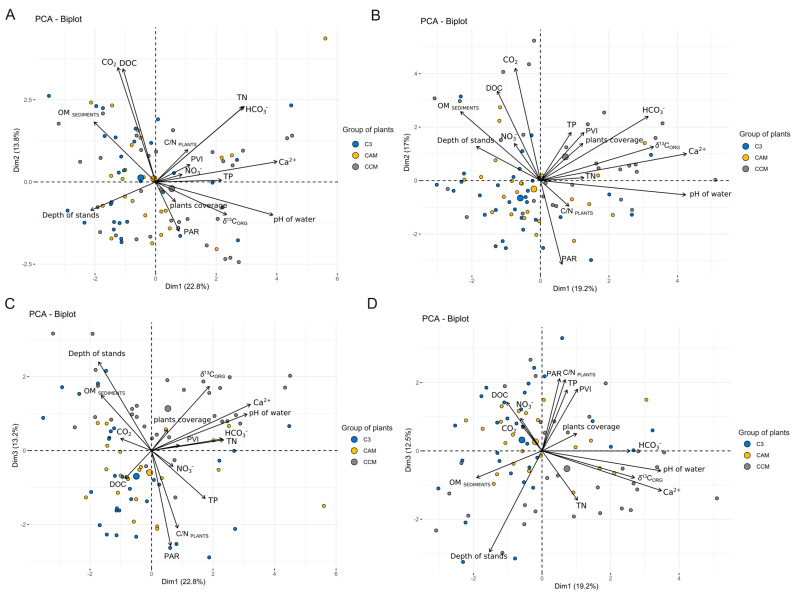
PCA analysis: (**A**)—ambient water variables and the δ^13^C of plants and other measured parameters (*n* = 85) and first and second dimensions, (**B**)—above sediment water variables, and δ^13^C of plants and other measured parameters (*n* = 85) and first and second dimensions, (**C**)—ambient water variables and the δ^13^C of plants and other measured parameters (*n* = 85) and first and third dimensions, and (**D**)—above sediment water variables, and δ^13^C of plants and other measured parameters (*n* = 85) and first and third dimensions. TN—total nitrogen and TP—total phosphorus, DOC—dissolved organic carbon, PAR—photosynthetic active radiation, PVI—percentage volume infested by plants, and OM_SEDIMENTS_—% of the OM in the sediments. The biggest circles indicated the centroids of each group.

Principal components analysis (PCA) was conducted for each type of water to reveal the primary relationships that significantly influenced the δ^13^C_ORG_. The first dimension in the ordination area of both analyses revealed a robust correlation with pH, affirming its substantial influence on δ^13^C_ORG_ values (Figure 3A–D). The relationships observed between δ^13^C_ORG_ with pH and Ca^2+^ were similar in both water types (Figure 3A–D). However, the explained variance of the three dimensions was marginally higher for the ambient water of surrounding plants (49.8%) compared with water from sediments (48.7%, Figure 3A–D). Notably, there was no distinct separation between the investigated groups of photosynthesis pathways and the CCMs group on the PCA graphs. Representatives of all groups were distributed across almost all ordination locations. However, in the plot where the first and the third dimensions were plotted, the sites of the CCM group were more concentrated in the upper-right corner than C3 plans, located more in the down-left corner (Figure 3C). In the case of the above sediment water, the most aggregated sites from the CCM group were also present in the down-right corner. Still, the other two groups were placed in the upper-left corner (Figure 3D). Moreover, the PERMANOVA analysis shows statistical differences between investigated groups (F = 5.08, *p* < 0.001; F = 5.09 *p* < 0.001), which explains 11.03% and 11.05% of group variances in the ambient and above-sediment water datasets, respectively. 

## 3. Discussion

As highlighted in the introduction section, aquatic plants remarkably differ in their values of *δ*^13^C_ORG_, which could be influenced by photosynthesis. Moreover, several abiotic factors, such as the isotopic signature of sources used in biosynthesis processes, are highly essential [8,13]. Several studies emphasize the influence and impact of different forms of carbon in shaping the *δ*^13^C_ORG_ as signalized in the Introduction section [3,4,5,6,7,8,9,10,11]. However, our results show notable differences between the CCMs group of plants with the ability to use HCO_3_¯ and the CAM and C3 groups of investigated plants in specific Lobelia lakes ecosystems (Figure 1). Higher *δ*^13^C_ORG_ values were recorded mainly for plants collected in the neutral and alkaline sites in the CCMs group of investigated lakes (Figure S1 in Supplementary Materials). This observation confirms the presence of HCO_3_^−^ users in this group. 

The distinction between photosynthetic pathways in aquatic plants based on *δ*^13^C_ORG_ values is not as straightforward as in terrestrial plants, where *δ*^13^C_ORG_ values can differentiate C3 and C4 plants [15,16,32]. In Lobelia lakes, the CAM and C3 plants obtained similar results of *δ*^13^C_ORG_ in terms of high ^12^C-enriched values than the CCMs group. However, our results suggested that the CCM plant groups of HCO_3_¯ users in Lobelia lakes ecosystems might be easily identified in the presence of CAM species, which are commonly found in these types of lakes. 

In that study, we reported that the highest *δ*^13^C_ORG_ values were found in elodeids species represented by *E*. *canadensis* and *M*. *alterniflorum* (grouped as CCM plants); moderate values were recorded for charophytes (*C*. *globularis* and *N*. *flexilis,* also included in CCMs plants group), significantly lower values were found for isoetids (*L. dortmanna* and *L. natans*—both included in C3 plants group and *L*. *uniflora* and *I*. *lacustris* included in CAM group of plants), and the lowest values were found for mosses (included in C3 plants group for details, please see Supplementary Tables S1 and S2).

These reported results clearly distinguished the species that efficiently used HCO_3_¯ as a carbon source during photosynthesis in higher pH environments and those that cannot use this form of carbon source (i.e., isoetids and mosses). Furthermore, when we divided the investigated photosynthesis and CCM groups based on water pH in their occurrence sites, we observed that in the CCM group, the *δ*^13^C_ORG_ values were significantly higher in the alkaline sites compared with acidic and neutral ones (Figure S1 in Supplementary Materials). It reaffirmed the pH dependency of *δ*^13^C_ORG_ values in the CCM group.

Additionally, our results distinctly illustrated that the values of CCMs in alkaline and neutral sites were significantly higher than those reported in pH groups for C3 and CAM photosynthesis (Figure S1 in Supplementary Materials). Conversely, C3 and CAM exhibited the highest *δ*^13^C_ORG_ values in acidic pH (Figure S1 in Supplementary Materials). Notably, the elevated high *δ*^13^C_ORG_ values in acidic sites were observed, especially in the case of the CAM group. These higher *δ*^13^C_ORG_ values are probably linked to this plant’s better growth conditions or might result from CO_2_ limitation during intensive photosynthesis, leading the plant to utilize ^13^CO_2_ more frequently for photosynthesis. The minor variation of *δ*^13^C_ORG_ values between pH classes inside the CAM group was probably caused by the lack of differentiation of the carbon source. This is perhaps due to most sediment CO_2_ users belonging to this group [28,33]. We also believe that the microbiological relationships with the roots significantly contribute to establishing the *δ*^13^C_ORG_ values of CO_2_ sediment-dependent isoetid plants. The effective mineralization of the autochthonous and allochthonous ^12^C-enriched material in Lobelia lakes might be crucial to ^12^CO_2_ availability. Moreover, in the limited literature available, it has been indicated that *L*. *uniflora*, *I*. *lacustris*, and *L*. *natans* tend to favor slightly acidic conditions [19,34,35]. Furthermore, these two species mentioned above develop big and strong roots, which oxidate the sediment and enhance the faster OM mineralization [19], which might cause the greater release of ^12^CO_2_ in a neutral and alkaline environment where also the mineralization of OM accelerates in comparison to acidic environmental conditions [36].

Despite several attempts to assess how individual environmental variables influence the diversity of plants *δ*^13^C_ORG_ of the considered groups of photosynthetic pathways and mechanisms, no specific dominant influence of particular environmental factors was evident. Still, combining several factors seems more critical due to the complexity of aquatic plants *δ*^13^C_ORG_ setting. The PCA analysis did not reveal distinct relationships assigned to particular groups regarding environmental changes considered in the presented research. However, significant dependencies and associations for all considered groups seemed to be linked to the pH gradient and Ca^2+^ concentration, as demonstrated by PCA analyses conducted for ambient and sedimentary waters (Figure 3). In this PCA analysis, we observe significant relationships with the light conditions in the stands from which the plant material was collected, particularly noting the negative relationship between DOC and PAR—photosynthetic active radiation, and its impact on *δ*^13^C_ORG_. This relationship can be interpreted in two primary ways. Firstly, as DOC concentration increases, *δ*^13^C_ORG_ decreases, particularly in Lobelia lakes surrounded by coniferous forests (see Supplementary Materials Figure S2), contributing to an increased inflow of humic substances. This influx reduces PAR by absorbing and scattering light, leading submerged plants to favor ^12^C over ^13^C, thus enhancing ^12^C enrichment in *δ*^13^C_ORG_. Secondly, accelerated eutrophication increases algae biomass in more alkaline Lobelia lakes with urban or agricultural catchments, reducing light transparency and, consequently, PAR. This diminished PAR reduces the plants’ demand for carbon sources, prompting them to enhance their discrimination between ^12^C and ^13^C, resulting in ^12^C enrichment in *δ*^13^C_ORG_. The PCA biplots (Figure 3C,D) support these interpretations, showing DOC and PAR vectors in opposite directions, indicating a negative relationship, and the *δ*^13^C_ORG_ vector aligning with reduced PAR. Environmental factors such as NO_3_¯, TP, and TN correlated positively with PCA dimensions and further aligned with eutrophication impacts [37]. Moreover, our previous study also signaled those *δ*^13^C_ORG_ dependencies with light conditions, where we compared isotopic signals of one charophyte species from Lobelia lakes with those of more hardwater lakes with charophytes [11]. 

Our study revealed the differences between C3, CAM, and CCM groups regarding *δ*^13^C_ORG_. The PCA analysis and heat map of correlations with environmental conditions showed a significant relationship between *δ*^13^C_ORG_ and the Ca^2+^ concentration and pH of the water, which was found solely in the CCMs group. The PCA analysis demonstrated the impossibility of pointing out the main environmental variables influencing the *δ*^13^C_ORG_ in a specific group of photosynthesis types and CCMs. Hence, we postulate that for studies intending to utilize *δ*^13^C_ORG_ values of macrophytes, it is crucial to focus more on each ecological group separately.

Despite this limitation, it should be noted that the data presented for plants in the investigated photosynthesis groups here clearly differs and might help identify the succession in the Lobelia lakes starting from the decline of mosses and reduction in isoetids present, which are replaced by elodeids [29]. The isotopic analyses of aquatic plants, even those not identified to the species or ecological groups, might help to determine the succession stage of the Lobelia lakes vegetation. This might be helpful with the recognition of the Lobelia lakes’ status and thus implement the management plans for better protection of those rare and specific ecosystems.

## 4. Materials and Methods

### 4.1. Study Sites

**Figure 4:**
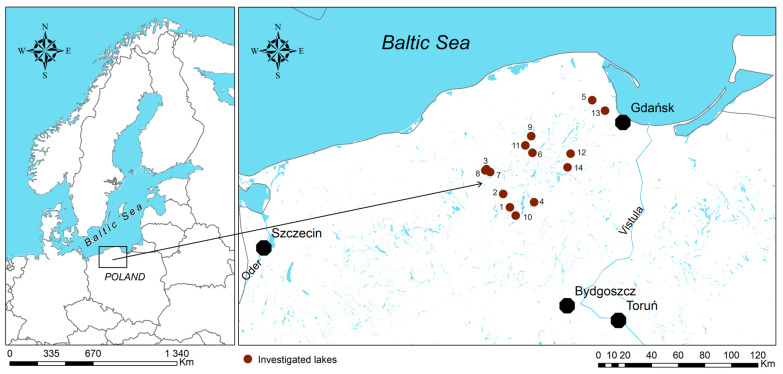
Localization of the investigated lakes. The numbers correspond to the lake’s order provided in Table 1.

The study included 14 Lobelia lakes investigated in the middle (July) of the growing 2020 season (Figure 4). Those lakes were diverse in terms of several physicochemical and morphometrical parameters (Table 1). We focused on collecting plant species from different photosynthesis pathways in the field. Thus, in this paper, we grouped the ten investigated species into three groups based on their photosynthetic pathways: C3 (i.e., *L*. *dortmanna* L, *L*. *natans*, *Fontinalis antipyretica* Hedw., and *Sphagnum denticulatum* Brid.), CAM (i.e., *L*. *uniflora*. and *I*. *lacustris*), and CCMs group (i.e., *M*. *alterniflorum*., *E*. *canadensis*, *Nitella flexilis* (L.) AG., and *Chara globularis* Thuiller as the charophytes are also included to CCMs group [38]).

### 4.2. Field Study

At each plant study site (*n* = 80), a total of 85 plant samples (*n* = 85) were collected for further *δ*^13^C_ORG_ analyses. These collections were performed by an experienced SCUBA diver, typically gathering ten individuals of each plant species (in total, about 85 × 10 = 850 individuals). Before collecting the plant, field measurements were taken from a boat. This involved pH measurements using a YSI 650 MDS equipped with a Multiparameter Water Quality Sonde 6600 V2 (Yellow Springs, OH, USA). Photosynthetic active radiation (PAR) was measured using a Licor LI-250 Light Meter (LI-COR Environmental GmbH, Bad Homburg, Germany) and expressed here as a percentage of the light reaching the water surface just above the plants. Depth measurements of the plant stands were also recorded. Moreover, a diver collected two plastic 0.5 L bottles of water from the plants’ surroundings (*n* = 80). The next set of water samples was taken just above the sediment (*n* = 80). These water samples were intended for further chemical laboratory analyses. Additionally, the percentage volume infested by plants (PVI) was calculated, representing the results of the percentage coverage of the investigated plants multiplied by their height based on an average of five measurements and divided by the depth at which the patch developed, following a method described in the study of Pełechaty et al. [39]. Furthermore, before conducting our research and collecting the plants and sediments, we obtained the necessary permits from the Regional Director for Environmental Protection in Gdansk, Poland (for further details, see Tables S1 and S2 in the Supplementary Materials).

### 4.3. Laboratory Variable Analysis of Water Collected in the Field

In the collected water samples from the plant’s surroundings (*n* = 80) and just above sediments (*n* = 80), we assessed the concentration of dissolved forms of inorganic carbon (DIC—dissolved inorganic carbon: CO_2_, HCO_3_^−^, and CO_3_^2−^) in the water by titration. The calcium concentration (Ca^2+^) was determined using a complexometric method with disodium edetate in the presence of calconcarboxylic acid sodium salt as an indicator. Concentrations of NO_3_^−^, TN (total nitrogen), and TP (total phosphorus) were determined with photometric methods using the MERCK Spectroquant cuvette test on the UV–VIS spectrophotometer (Aquamate, Thermo Electron Corporation, Waltham, MA, USA). Therefore, TP analysis was performed after mineralizing water samples in a mixture of acids, sulfuric, and nitric in a 2:1 proportion in the microwave digestion system Mars 5 CEM (Matthews, NC, USA). DOC was measured with a UV–VIS spectrophotometer at a wavelength of 330 nm. To determine the sediment OM, we calculated it based on the difference in dry sediment weight before and after combustion at 550 °C (Thermolyne 62700 muffle furnace, Waltham, MA, USA). The C/N ratios were calculated by obtaining the C and N percentages in the sediment and plant samples (EA VarioMicro Cube, Elementar, Langenselbold, Germany, and Flash Smart EA, Thermo Scientific, Waltham, MA, USA, respectively). Merdalski et al. [40] and Pronin et al. [11] gave more detailed descriptions of the applied method. 

### 4.4. The Analyses of Plant Material, Including δ^13^C_ORG_ Analyses

The collected plant samples were washed in the field, and the epiphytes and other contaminations (sand, sediments, and others) were removed and washed using deionized water. The plant samples were dried at 60 °C for 48 h and stored dry. In the laboratory, dry plant samples were crushed into a powder using a mixer mill (MM 400 Retsch, Haan, Germany) or agate mortar for smaller-sized plants. Given the negative HCl test check results, indicating the absence of carbonates in prepared material, we conducted bulk δ^13^C_ORG_ analyses of plants from the field in 85 powdered samples (triplicated as a standard laboratory procedure; in total, 255 samples) in the GISMO platform, Biogéosciences laboratory of the University of Burgundy (Dijon, France) on an elementar analyzer Flash Smart EA (Thermo Scientific, Waltham, MA, USA) coupled to a Delta V stable isotope ratio mass spectrometer (Thermo Scientific, Waltham, MA, USA). Standard USG40 (glutamic acid, δ^13^C = −26.39‰) and the standard Wheat Flour B2157 (Elemental Microanalysis, Okehampton, UK) certified reference materials were used for calibration and control. δ^13^C values were expressed as a notation in ‰ relative to the Vienna Peedee Belemnite (V-PDB). The precision of the analysis was confirmed through the external reproducibility testing of replicate standard analyses of USG40, and the B2157 standards were better than ±0.15‰ for δ^13^C (2σ).

### 4.5. Statistical Analysis

The normality of the distributions for the analyzed δ^13^C values of plant OM, as well as the water physicochemical parameters and other environmental variables, was evaluated with the Shapiro–Wilk test using the R.4.2.1 software [41]. The results indicated that most analyzed parameters, especially δ^13^C, did not follow a normal distribution. Consequently, non-parametric analyses were applied. The Kruskal–Wallis test was used to compare the values of the δ^13^C of plants with different photosynthesis pathways. Dunn’s post hoc test was performed to identify the differences between established groups of plants [42] using the Dunn test package in R.4.2.1 software. For all statistical tests, the *p* < 0.05 was accepted. Furthermore, Spearman rank correlations were used to investigate the relationships between δ^13^C_ORG_ of all plants and the water physicochemical parameters and other environmental variables measured for two types of investigated water. These analyses were performed using the R.4.2.1 software and visualized using the ggplot2 [43] and corrplot [44] and ggstatsplot [45] packages. Finally, we conducted a principal component analysis (PCA) on the isotopic data and other investigated parameters for ambient and above sediment waters separately based on the photosynthesis pathway groups of the investigated plants using the FactoMineR package [46] to check which variables have a crucial impact on δ^13^C_ORG_ of all plants. PCA results were visualized using the factoextra package [47]. Moreover, the PERMANOVA analysis for the two separate datasets of ambient and above sediment waters was performed to check if there are differences between the investigated plants group. This analysis was conducted using the R.4.2.1 software and vegan package [48]. 

## 5. Conclusions

This study reveals distinct differences in *δ*^13^C_ORG_ values among C3, CAM, and CCM aquatic plants in northern Polish Lobelia lakes. CCM plants exhibit higher *δ*^13^C_ORG_ values due to using ^13^C-enriched HCO_3_^−^, particularly in neutral to alkaline conditions. CAM species show slightly higher *δ*^13^C_ORG_ values than C3 plants, especially in low pH environments. Increased DOC from coniferous forests reduces PAR by absorbing and scattering light, leading to enhanced ^12^C enrichment in *δ*^13^C_ORG_. Eutrophication in more alkaline lakes further decreases light transparency and PAR, affecting carbon source utilization by submerged plants.

PCA analysis highlights the significant positive influence of pH and Ca^2+^ on *δ*^13^C_ORG_ values, with environmental variables like NO_3_¯, TP, and TN also playing crucial roles. The differentiation of photosynthetic pathways based on *δ*^13^C_ORG_ values provides valuable insights into carbon cycling and the adaptation of aquatic plants to environmental changes. This knowledge is essential for managing and conserving unique ecosystems such as Lobelia lakes. Our findings emphasize the complexity of carbon isotope dynamics and the need for future research on the interplay of environmental factors affecting *δ*^13^C_ORG_ values.

In conclusion, the study underscores the importance of *δ*^13^C_ORG_ values as indicators of photosynthetic pathways and environmental conditions and might be helpful for conservation strategies and ecosystem management efforts.

## Figures and Tables

**Table 1:** The characteristics of the investigated lakes and measured physiochemical variables in all sites in the lake. In the table, the values after ± indicated standard deviation from the average values for all investigated sites in the lake.

Lake	Lake Geographical Coordinates Converted	Lake Surface	Ca^2+^	TN	TP	CO_2_	HCO_3_^−^	DIC	OM	pH	Cond.
		[km^2^]	[mg/L]	[mg N/L]	[mg P/L]	[mg C/L]	[mg C/L]	[mg C/L]	[%]		[µS cm^−1^]
1. Linowskie	53°46′38.27″ N, 17°21′49.00″ E	0.14	1.73 ± 0.08	1.06 ± 0.08	0.02 ± 0.01	2.07 ± 0.61	5.54 ± 0.71	7.61 ± 0.74	4.02 ± 2.73	5.15 ± 0.24	22.83 ± 0.25
2. Krasne	53°52′5.31″ N, 17°16′52.93″ E	0.29	2.28 ± 0.12	0.83 ± 0.33	0.03 ± 0.01	1.58 ± 0.29	7.89 ± 0.57	9.47 ± 0.77	15.76 ± 1.60	5.90 ± 0.20	24.57 ± 0.32
3. Smołowe	54°1′56.96″ N, 17°4′48.84″ E	0.36	2.31 ± 0.22	0.70 ± 0.09	0.03 ± 0.01	1.90 ± 0.73	3.96 ± 0.28	5.79 ± 0.66	10.40 ± 15.33	6.21 ± 0.08	26.29 ± 0.57
4. Moczadło	53°48′49.87″ N, 17°38′6.39″ E	0.04	2.72 ± 0.09	0.66 ± 0.07	0.03 ± 0.02	1.20 ± 0.13	3.84 ± 0.63	5.04 ± 0.65	1.70 ± 1.16	6.24 ± 0.25	30.08 ± 1.19
5. Piasek	54°0′46.71″ N, 17°7′4.50″ E	0.41	3.74 ± 0.27	0.10 ± 0.14	0.06 ± 0.07	1.23 ± 0.24	6.08 ± 1.45	7.31 ± 1.48	6.74 ± 5.77	6.79 ± 0.19	50.10 ± 0.1.47
6. Kamień	54°1′39.39″ N, 17°3′51.89″ E	0.5	5.24 ± 0.15	0.54 ± 0.06	0.02 ± 0.01	1.49 ± 0.14	7.47 ± 0.37	8.96 ± 0.41	11.72 ± 11.94	6.79 ± 0.26	50.02 ± 3.86
7. Łąkie	54°8′53.37″ N, 17°36′13.05″ E	0.22	4.30 ± 0.19	0.56 ± 0.12	0.02 ± 0.02	1.97 ± 0.62	5.86 ± 0.79	7.83 ± 1.09	17.08 ± 20.68	6.69 ± 0.20	49.41 ± 1.12
8. Zawiad	54°30′37.76″ N, 18°17′11.06″ E	0.17	3.15 ± 0.19	0.77 ± 0.13	0.09 ± 0.06	0.94 ± 0.20	5.44 ± 2.58	6.42 ± 2.64	2.80 ± 1.92	7.16 ± 0.49	39.92 ± 0.35
9. Okoń Duży	53°43′14.83″ N, 17°25′34.12″ E	0.12	4.38 ± 0.05	1.23 ± 0.42	0.06 ± 0.07	1.36 ± 0.55	5.16 ± 0.53	6.52 ± 0.83	3.17 ± 1.74	7.44 ± 0.33	46.83 ± 2.58
10. Obrowo Małe	54°15′40.13″ N, 17°35′12.42″ E	0.09	2.95 ± 0.33	0.45 ± 0.06	0.09 ± 0.05	1.01 ± 0.14	4.22 ± 0.59	5.23 ± 0.57	6.02 ± 6.22	7.44 ± 0.55	30.14 ± 0.57
11. Jeleń	54°11′52.46″ N, 17°31′14.75″ E	0.81	6.42 ± 0.19	0.65 ± 0.34	0.07 ± 0.05	1.27 ± 0.78	4.89 ± 1.41	7.31 ± 1.33	5.16 ± 3.84	8.14 ± 0.40	56.41 ± 0.95
12. Dobrogoszcz	54°8′49.62″ N, 18°2′30.012″ E	0.54	11.95 ± 0.21	1.34 ± 0.10	0.04 ± 0.02	1.52 ± 0.33	8.33 ± 0.81	9.86 ± 0.72	1.04 ± 0.04	8.30 ± 0.10	193.50 ± 0.98
13. Osowskie	54°26′24.85″ N, 18°26′13.28″ E	0.28	15.30 ± 0.18	2.55 ± 0.64	0.15 ± 0.10	1.95 ± 0.47	10.30 ± 030	12.25 ± 0.77	2.22 ± 0.50	8.75 ± 0.65	142.95 ± 0.09
14. Zakrzewie	54°3′14.86″ N, 18°0′39.54″ E	0.10	10.37 ± 0.06	0.90 ± 0.07	0.02 ± 0.01	0.32 ± 0.52	6.41 ± 1.04	7.49 ± 1.24	0.80 ± 0.30	9.05 ± 0.08	69.12 ± 0.61

TN—total nitrogen and TP—total phosphorus, DIC—dissolved inorganic carbon OM—% of the organic matter in the sediments, Cond.— conductivity.

## Data Availability

Most of the data generated or analyzed during this study are included in this article and its Supplementary Materials. The authors make the rest of the included data available upon reasonable request.

## Supplementary Materials

The following supporting information can be downloaded at https://www.mdpi.com/article/10.3390/plants13172529/s1: Figure S1: Comparison of the *δ*^13^C isotopic values of OM from different plants CCMs group, C3, and CAM photosynthesis pathways groups concerning the water pH in the sites from which the plants were collected: Acidic pH < 6.5, Neutral pH > 6.5 and <7.2, Alkaline pH > 7.2; Bigger case and lower case letters if different means the statistical significance *p* < 0.05 Dunn posthoc test after Kruskal–Wallis test; Figure S2: The catchment land use characteristics of investigated Polish Lobelia lakes; Table S1: The characteristics of the study sites with the raw data of measured variables and ambient water parameters; and Table S2. The characteristics of the study sites with the raw data of measured variables and above sediment water parameters.
