# Supplementary material for: Lobelia Lakes’ Vegetation and Its Photosynthesis Pathways Concerning Water Parameters and the Stable Carbon Isotopic Composition of Plants’ Organic Matter
Source: Plants (Basel). 2024 Sep 9;13(17):2529. doi: 10.3390/plants13172529 (PMC11396979; doi:10.3390/plants13172529)
Supplement: Supplementary file 1 [file plants-13-02529-s001.zip › Supplementary Materials Figure S1 and S2.pdf]

Lobelia Lakes' Vegetation and Its Photosynthesis Pathways Concerning Water Parameters and the Stable Carbon Isotopic Composition of Plants' Organic Matter

Eugeniusz Pronin[1*], Krzysztof Banaś[1], Rafał Chmara[1], Rafał Ronowski[1], Marek Merdalski[1], Anne-Lise Santoni[2], Olivier Mathieu[2]

1 Department of Plant Ecology, Faculty of Biology, University of Gdansk, Gdańsk80-309 Gdansk, Poland; krzysztof.banas@ug.edu.pl (K.B.); rafal.chmara@ug.edu.pl (R.C.); rafal.ronowski@ug.edu.pl (R.R.); marek.merdalski@ug.edu.pl (M.M.)

2 Biogéosciences, UMR 6282 CNRS, Université Bourgogne Franche-Comté, F-21000 Dijon, France; anne-lise.santoni@u-bourgogne.fr (A.-L.S.); olivier.mathieu@u-bourgogne.fr (O.M.)

* Correspondence: eugeniusz.pronin@ug.edu.pl; Tel.: +48-58-523-61-27

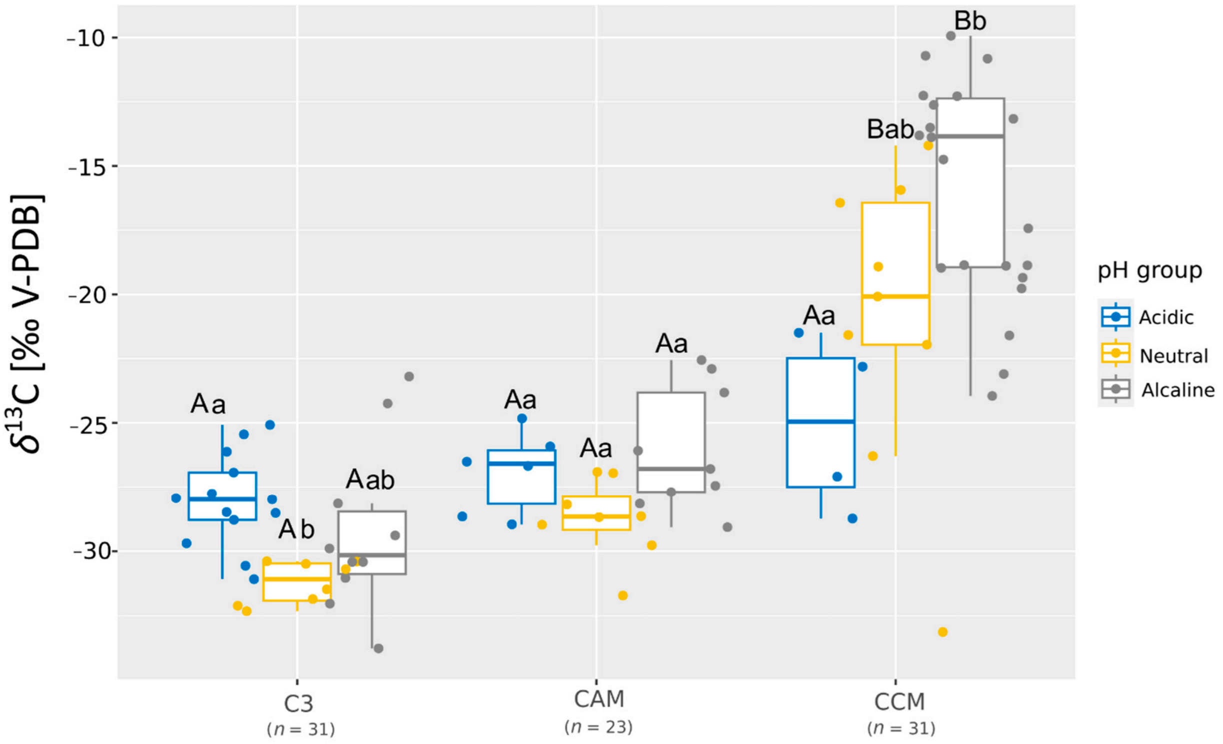

Figure S1. Comparison of the $\delta^{13}C$ isotopic values of OM from different plants CCMs group, C3, and CAM photosynthesis pathways groups concerning the water pH in the sites from which the plants were collected: Acidic pH < 6.5, Neutral pH >6.5 and <7.2, Alkaline pH > 7.2; Bigger case and lower case letters if different means the statistical significance $p < 0.05$ Dunn posthoc test after Kruskal–Wallis test.

Forests catchment

Krasne Lake

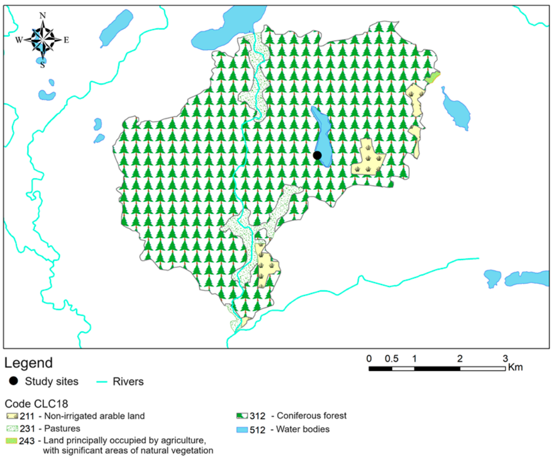

Linowskie Lake

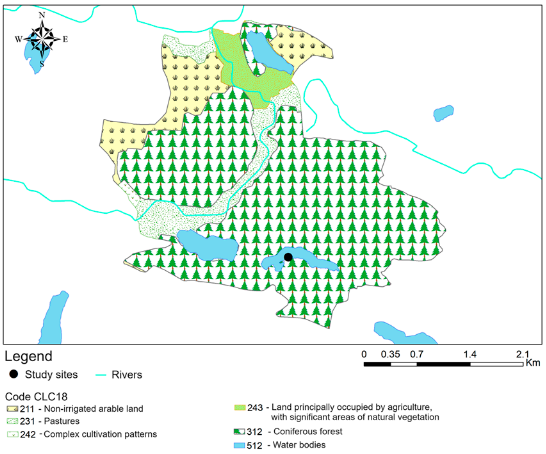

Okoń Duży Lake

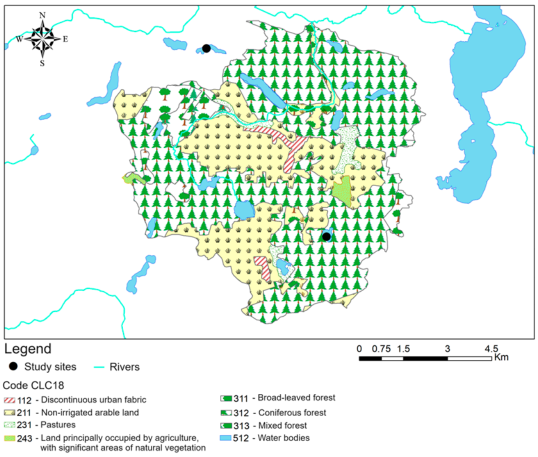

Zakrzewie Lake

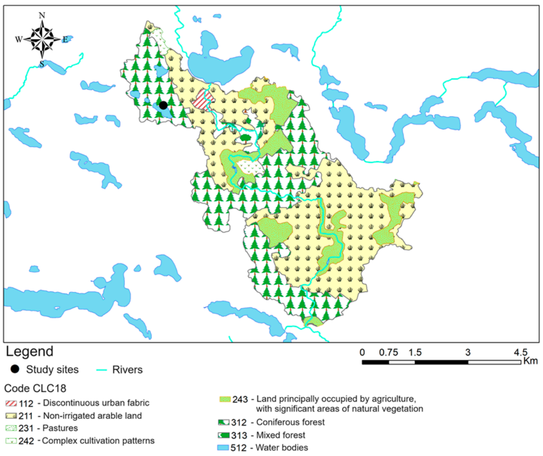

Obrowo Małe Lake

Zawiad Lake

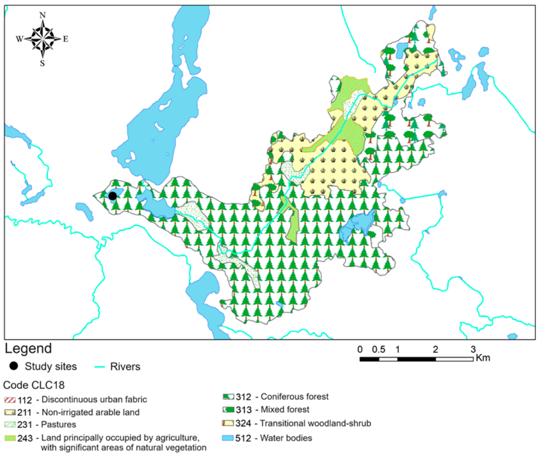

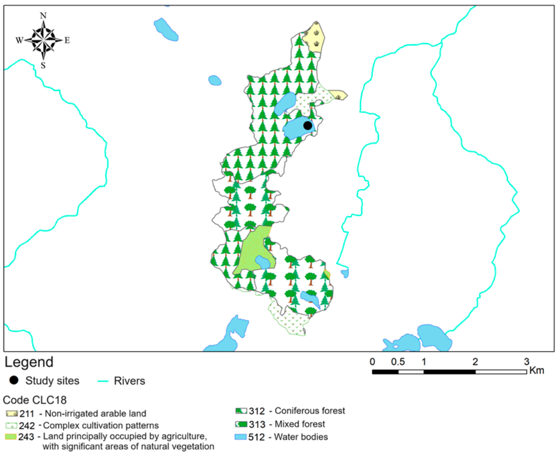

Moczadło Lake

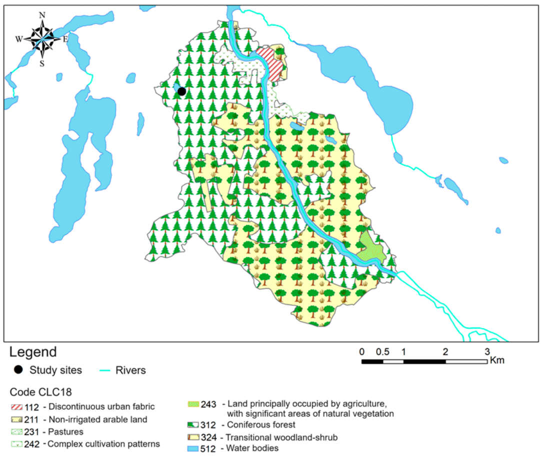

Forests and arable land with some urban fabric.

Dobrogoszcz Lake

Osowskie Lake

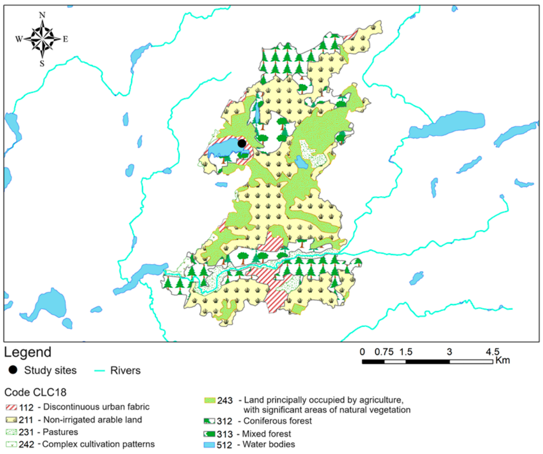

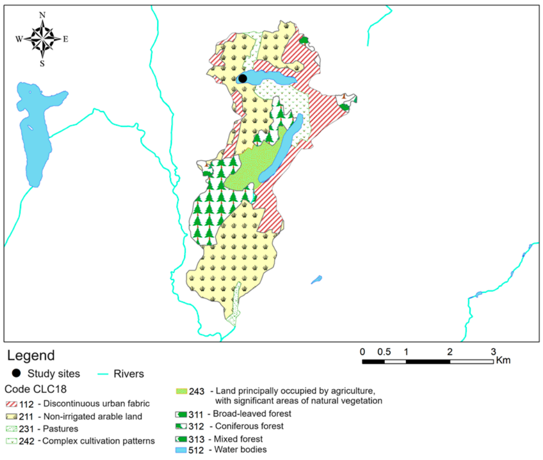

Arable land with forests

Jeleń Lake

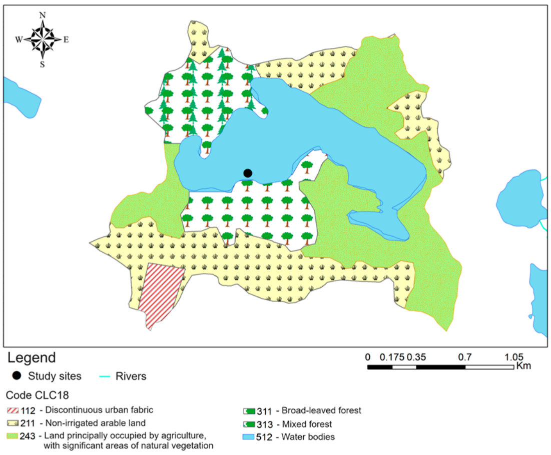

Kamień & Smołowe Lake

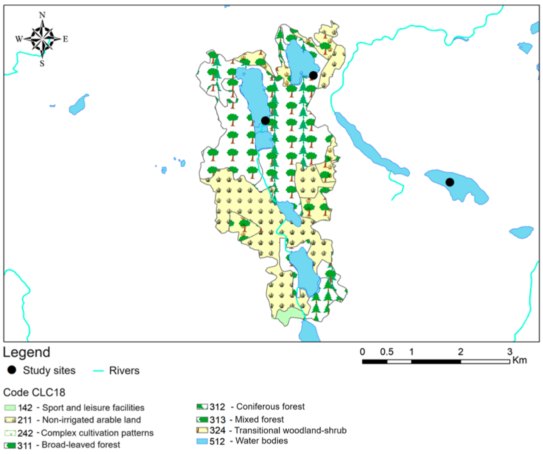

Piasek Lake

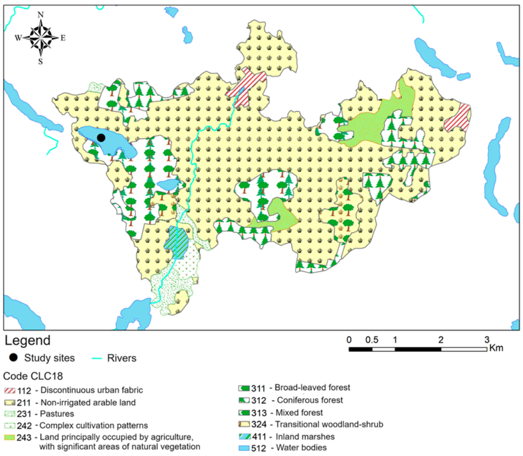

Łąkie Lake

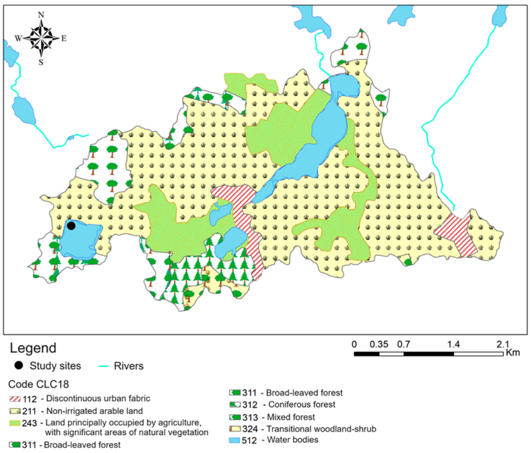

Figure S2. The catchment land use characteristics of investigated Polish Lobelia lakes
